# Arylamine *N*-Acetyltransferase 1 Activity is Regulated by the Protein Acetylation Status

**DOI:** 10.3389/fphar.2022.797469

**Published:** 2022-01-27

**Authors:** Raúl A. Salazar-González, Mark A. Doll, David W. Hein

**Affiliations:** Department of Pharmacology and Toxicology, Brown Cancer Center, University of Louisville, Louisville, KY, United States

**Keywords:** lysine acetylation, arylamine N-acetyltransferase 1, histone deacetylases inhibition, Sirtuin, breast cancer

## Abstract

Arylamine *N*-acetyltransferase 1 (NAT1) is a drug metabolizing enzyme that influences cancer cell proliferation and survival, especially in breast cancer. Lysine-acetylation is an important Post-Translational Modification (PTM) in the regulation of diverse cellular processes. Histone deacetylases (HDACs) and Sirtuins (SIRT) may have an important role on the NAT1 acetylation status, affecting its catalytic capacity and having an impact on the downstream functions of this protein. The aim of the present work is to investigate the acetylation status of NAT1 in human breast cancer. Breast cancer cell lines MDA-MB-231 (ER-, PR-, HER2-) and ZR-75-1 (estrogen receptor+, PR+, HER2+) were cultured in the presence of HDAC inhibitors (SAHA, TSA) or Sirtuin inhibitors (AGK2, EX527, Sirtinol). Under these conditions, NAT1 protein and gene expression as well as enzymatic activity were quantified. Acetylation of NAT1 protein was evaluated following an immunoprecipitation protocol and acetyl-Lysine quantification. *Sirt1* and *Sirt2* knockdown were performed and NAT1 protein and NAT1 mRNA expression and catalytic activity were quantified. The treatment of MDA-MB-231 or ZR-75-1 cells with increasing HDAC inhibitors resulted in 2 to 15-fold upregulation in NAT1 message expression. Finally, the catalytic activity of NAT1 in the presence of HDAC inhibition increased 2-fold. Conversely, the inhibition of Sirtuin activity did not cause significant changes in NAT1 message but produced a significant decrease in NAT1 catalytic activity. NAT1 acetylation was higher in the cells treated with HDAC inhibitors, as well as Sirtuin inhibitors. Finally, silencing of *Sirt1* and *Sirt2* genes by siRNA transient knockdown of each or both genes resulted in reduction of NAT1 protein expression and catalytic activity. The use of HDAC and Sirtuin inhibitors has been demonstrated as a promising powerful therapeutic alternative in various cancers. These inhibitors can significantly attenuate tumor burden by limiting tumor growth and metastasis. These compounds can also induce DNA damage, cell cycle arrest, apoptosis, and autophagy to promote cancer cell death. Several studies have shown that NAT1 is upregulated in cancer cells. The results of the present study show that the acetylation status of NAT1 is an important factor that might have a relevant role in the progression of cancer.

## Introduction

Breast cancer has the highest incidence among women worldwide, it is estimated that in 2021, about 30% of newly diagnosed cancers will be breast cancer (American Cancer Society, 2021). As of 2021, breast cancer became the most common cancer globally, accounting for 12% of all new annual cancer cases worldwide according to the World Health Organization (WHO, 2021).

Human arylamine *N*-acetyltransferase 1 (NAT1) is subject to genetic polymorphism in human populations and metabolizes many xenobiotics and carcinogens by catalyzing the transfer of an acetyl group from acetyl-CoA to the primary amine or N-hydroxyl group of these compounds (Hein et al., 2000; Hein, 2002). In the presence of folate, this enzyme can also hydrolyze acetyl-CoA to CoA (Laurieri et al., 2014; Stepp et al., 2017) and regulate the intracellular levels of acetyl-CoA (Stepp et al., 2019). Several reports have demonstrated the upregulation of NAT1 in breast cancer is associated with estrogen receptor (ER) expression (Carlisle and Hein, 2018; Minchin and Butcher, 2018; Zhang et al., 2018). Furthermore, NAT1 overexpression in primary breast cancer tumors is higher in ER^+^ compared to ER^−^ primary tumors (Carlisle and Hein, 2018).

Accurate control of protein function is of high importance for the organization and function of biological systems. Reversible post-translational modifications (PTM) offer a sophisticated mechanism to control protein function. A key advantage of PTMs is that they can be dynamically regulated at a higher rate and consume less energy than protein turnover. Lysine acetylation is a highly conserved PTM occurring in prokaryotes and eukaryotes. Early studies first described the acetylation mechanism in histone proteins (Allfrey et al., 1964), later studies found that high-mobility group (HMG) proteins (Sterner et al., 1979) and tubulin (L'Hernault and Rosenbaum, 1983), also undergo acetylation. Discoveries like the acetylation of p53, the description of mammalian histone acetylases (HATs) and histone deacetylases (HDACs), and the development of potent deacetylase inhibitors (Verdin and Ott, 2015), paved the way for the field of non-histone protein acetylation. Recently, some studies have suggested that acetylation of NAT1 protein can regulate its enzymatic function. Alterations in Lysine 100 (K^100^) of NAT1, decreased the affinity of acetyl-CoA for the protein, suggesting the importance of this residue for the binding of acetyl-CoA to the active site (Minchin and Butcher, 2015). A subsequent study, found that K^100^ was associated with the binding of ATP to NAT1 (Minchin et al., 2018); and a more recent study by the same research group, acetylation of NAT1 was enhanced by the Sirtuin inhibitor nicotinamide but not by the histone deacetylase inhibitor trichostatin A. Co-transfection of cells with NAT1 and either SIRT1 or two significantly decreased NAT1 acetylation (Butcher et al., 2020).

In the present study, we investigated the contribution of Lysine deacetylases (KDAC) in the catalytic activity of NAT1 using two different breast cancer cell lines: MDA-MB-231 (ER-, PR-, HER2-) and ZR-75-1 (ER+, PR+, HER2+). We tested the ability of Sirtuin and HDAC inhibitors to impact the transcription, translation and function of NAT1. Using a different approach, we transiently knocked down (KD) Sirtuin 1/2 and evaluated the role of these specific proteins in the same experimental outcomes.

## Materials and Methods

### Cell Lines and Cell Culture

Breast cancer cell lines MDA-MB-231 and ZR-75-1 were obtained from the American Type Culture Collection (ATCC, Manassas, VA, United States). MDA-MB-231 is estrogen receptor-negative, progesterone receptor-negative and HER2-negative whereas ZR-75-1 is estrogen receptor-positive, progesterone receptor-positive and HER2-positive. Cells were cultured In Dulbecco’s Modified Eagle Medium (DMEM) high glucose (4.5 g/L) or Roswell Park Memorial Institute (RPMI) 1640 culture media, respectively (Invitrogen, Waltham, MA, United States), supplemented with 10% Fetal Bovine Serum (Hyclone, Logan, UT, United States), 1% Pen/Strep (Hyclone, Logan, UT, United States), and 2 mM L-Glutamine (Corning, Corning, NY, United States). Cells were grown and maintained in a humidified incubator set at 37°C with 5% CO_2_.

### Deacetylation Pathway Evaluation

In order to test the effect of deacetylase proteins on NAT1 *N*-acetylation, we used the selective SIRT1 inhibitor 6-chloro-2,3,4,9-tetrahydro-1H-carbazole-1-carboxamide (EX527), selective SIRT2 inhibitor 2-cyano-3-[5-(2,5-dichlorophenyl)-2-furanyl]-N-5-quinolinyl-2-propenamide (AGK2), and selective SIRT1 and SIRT2 inhibitor 2-[[(2-hydroxy-1-naphthalenyl)methylene]amino]-N-(1-phenylethyl)-benzamide (Sirtinol), (Selleck Chemicals, Houston, TX, United States). To test the role of HDAC proteins, we used total HDAC inhibitors Trichostatin A (TSA) and Vorinostat (SAHA) (Selleck Chemicals, Houston, TX, United States). We tested concentrations between 0 and 100 μM for all compounds in both cell lines. We tested different incubation times (data not shown), and report results following a 48-h incubation for all assays.

### Sirtuin and HDAC Activity

To test the effects from inhibition of Sirtuin and HDAC activity, MDA-MB-231 (1×10^5^) and ZR-75-1 (3×105) cells were plated into 24 well plates and allowed to adhere to the plate overnight. The next day, cells were treated with increasing concentrations, (0–100 µM) of the inhibitor for 48 h. Following incubation, the enzymatic activity of Sirtuin and HDAC proteins was quantified using the Sirtuin Activity kit (MilliporeSigma, St. Louis, MO, United States), according to manufacturer’s instructions. Briefly, after the appropriate time of inhibitor treatment, cells were lysed using the homogenization buffer provided and centrifuged at 16,000 × *g* for 20 min at 4°C. Supernatant was recovered ant kept on ice. Samples were normalized to the lowest protein concentration sample using the Bradford protein assay (Bio-Rad, Hercules, CA, United States). Then, 20 µL of diluted cell lysate were transferred into a 96 well plate, the activity reaction mix was prepared as recommended, and the reaction was incubated for 15 min at 37°C. Fluorescence was quantified at 360/528 nm Ex/Em using a BioTek Synergy HT (BioTek, Winooski, VT, United States) plate reader. Enzymatic activity is reported as a percent compared to untreated vehicle control (0.5% DMSO).

### mRNA Quantification

NAT1 mRNA levels were measured as described previously (Millner et al., 2012). Primers were designed to recognize only NAT1b expression. Briefly, total RNA was isolated cells lines using the RNeasy Mini kit (Qiagen, Hilden, GER) followed by removal of contaminating DNA by treatment with Turbo DNA-free (Ambion, Austin, TX, United States). cDNA was synthetized using the High-Capacity Reverse transcriptase kit (Life Technologies, Carlsbad, CA, United States) using 1 µg of total RNA in a 20 µL reaction following manufacturer’s instructions. Quantitative Reverse Transcription polymerase Chain Reaction (qRT-PCR) was used for the quantification of relative amount of *NAT1*, *SIRT1* or *SIRT2* mRNA in MDA-MB-231 and ZR-75-1 cells after drug treatment or siRNA KD assays. qRT-PCR reactions were done in the Step One Plus (Life Technologies, Carlsbad, CA, United States) containing 1 × final concentration of iTaq universal SYBR Green Supermix (Bio-Rad, Hercules, CA, United States) and 500 nm of each primer (Table 1) in a 20 µL reaction. β2-microglobulin (*B2M*) was used as housekeeping control to determine $\Delta C_{t}$ (gene of interest $C_{t}-B2M\;C_{t}$ ). $\Delta \Delta C_{t}$ was determined by the subtraction of the smallest $\Delta C_{t}$ and relative amounts of the gene of interest mRNA were calculated using $2^{-\Delta \Delta C_{t}}$ as previously described (Barker et al., 2006; Salazar-Gonzalez et al., 2018b).

**TABLE 1:** Sequences of the primers used for the mRNA transcript expression.

Primer	Sequence
*NAT1b* Forward	5′-CTGGTTGCCGGCTGA AATAAC-3′
*NAT1b* Reverse	5′-TCC​AAG​TCC​AAT​TTG​TTC​CTA GACT-3′
*SIRT1* Forward	5′-CAA​GAC​CAT​TCT​TCA​AGT​TTG​CA-3′
*SIRT1* Reverse	5′-GTG​ACA​GAG​AGA​TGG​CTG​GAA​TT-3′
*SIRT2* Forward	5′-GCC​CTC​GCC​AAG​GAA​CTC​TA-3′
*SIRT2* Reverse	5′-CCT​TCA​GCA​GGC​GCA​TGA-3′
*B2M* Forward	5′-AGT​CAA​CTT​CAA​TGT​CGG​ATG​GAT-3′
*B2M* Reverse	5′-CCT​GGA​GGC​TAT​CCA​GCG​TAC-3′

### *In Vitro N*-Acetylation NAT1 Activity

*In vitro* NAT1 enzymatic activity was quantified towards *p*-aminobenzoic acid (PABA). *N*-acetyl-PABA was quantified by High Performance Liquid Chromatography (HPLC) as previously described (Hein et al., 2006; Stepp et al., 2018). Briefly, cells were scaped from the plate and washed in ice-cold Phosphate Buffered Saline (PBS) and lysed in 20 mM sodium phosphate pH 7.4, 1 mM ethylenediaminetetraacetic acid (EDTA), 0.2% triton X-100, 1 mM dithiothreitol (DTT), 100 µM phenylmethanesulfonyl fluoride (PMSF), 1 μg/ml aprotinin, and 2 µM pepstatin A (MilliporeSigma, St. Louis, MO, United States). Cell lysate was centrifuged at 15,000 × g for 10 min, supernatant was recovered, and protein concentration was quantified as mentioned before. NAT1 enzymatic assay reactions containing 50 µL of suitable cell lysate, 300 µM PABA, and AcCoA (1 mM), were incubated at 37°C for 10 min. Reactions were terminated by the addition of 10 µL of 1 M acetic acid, then proteins were precipitated by centrifugation (15,000 × g for 20 min in the cold room) and supernatant was transferred to HPLC vials and injected onto a LiChrospher 100 RP-18 (125 mm × 4 mm; 5 µm) reverse phase column. Reactants and products were eluted with an Agilent 1260 Infinity II system (Agilent Technologies, Santa Clara, CA, United States). Separation of *N*-acetyl-PABA was achieved using a gradient of 96:4 of 20 mM sodium perchlorate pH 2.5: acetonitrile (ACN) to 88:12 20 mM sodium perchlorate:ACN over 3 min. Absorbance was recorded at 280 nm. Four independent measurements were performed in duplicate for each cell condition and cell line.

### Transient siRNA Transfection

Sirtuin gene knockdown (KD) was performed on both breast cancer cell lines; MDA-MB-231 and ZR-75-1 cells were plated at a low density, 2×10^5^ cells/well in 12-well plates and allowed to adhere to the plate overnight. The next day a transient transfection with a mix of custom siRNA (Table 2) was directed against *Sirt1* or *Sirt2* (25 nM total concentration) or 25 nM of scrambled non-targeting siRNA (scr siRNA) (Horizon Discovery, Waterbeach, United States) using Lipofectamine 3000 (Thermo Fisher Scientific, Waltham, MA, United States) according to manufacturer’s recommendations. After 48 h of siRNA transfection, cells were harvested, and transfection efficiency was assessed by quantification of the target gene mRNA expression. For functional studies, the siRNA transfection was carried out for 72 h, and cells were harvested and lysed for further experiments.

**TABLE 2:** Sequences of the custom SiRNA molecules used to Knockdown Sirtuin genes.

Gene	SiRNA sequence
*Sirt1*	5′-GGA​AAU​AUA​UCC​UGG​ACA​AUU-3′
*Sirt1*	5′-UGG​AAC​AGG​UUG​CGG​GAA​UUU-3′
*Sirt1*	5′-GGC​AAU​UAA​UGA​AGC​UAU​AUU-3′
*Sirt2*	5′-GGC​UGG​AAC​AGG​AGG​ACU​UUU-3′
*Sirt2*	5′-CCA​UCU​GUC​ACU​ACU​UCA​UUU-3′
*Sirt2*	5′-GGC​GAU​UCG​ACC​UAC​UUU-3′
Non- targeting (scr siRNA)	5′-ACC​AAA​UGU​ACA​GCU​GAU​U-3′

### NAT1 Protein Quantification

Cells were plated in 96-well black/clear bottom plates (Thermo Fisher Scientific, Waltham, MA, United States) and incubated overnight at 37°C and 5% CO_2_. Then, cells were treated with either Sirtuin or HDAC inhibitors or siRNA. After the appropriate incubation time, NAT1 protein expression was evaluated using an in-cell western (ICW) protocol as previously described (Stepp et al., 2019; Salazar-Gonzalez et al., 2020). Briefly, the cells were fixed using 3.7% paraformaldehyde and permeabilized on the plate using TBS +0.1% Triton X-100. Then, plates were blocked using 1 × fish gelatin (Biotium Inc., Fremont, CA, United States). Staining was done using rabbit monoclonal anti-NAT1 antibody (ab109114, Abcam, Cambridge, United Kingdom) and mouse monoclonal anti-β-actin, as endogenous control (A2228, Sigma-Aldrich, St. Louis, MO, United States) overnight in the cold room. The next day, plates were washed with TBS +0.1% Tween 20 (TBST) and incubated with secondary antibodies IRDye^®^ 800 CW goat anti-rabbit and 680RD goat anti-mouse (LI-COR Biosciences, Lincoln, NE, United States) at room temperature for 1 hour. Finally, plates were washed with TBST and scanned using an Odyssey imaging system (LI-COR Biosciences, Lincoln, NE, United States). NAT1 protein expression was normalized to β-actin content in each well.

### *In vitro* NAT1 Deacetylation Assay

MDA-MB-231 or ZR-75-1 cells were plated in 12-well plates, at a density of 2 × 10^5^ cells/well and allowed to attach to the plate overnight. The next day cells were treated with either increasing concentrations of Sirtuin inhibitors (AGK2, EX527, Sirtinol) or HDAC (SAHA, TSA) inhibitors or a SiRNA transfection cocktail as described above. After 48 h of drug treatment, plates were recovered and the cells were harvested for NAT1 activity assays, NAT1 mRNA and protein expression quantification as described above. For the siRNA transfection experiments, after 72 h of transfection, cells were harvested and processed as described above for the cells treated with inhibitors.

### Immunoprecipitation

In order to assess the acetylation status of NAT1 protein, MDA-MB-231(6 × 10^5^) and ZR-75-1 (3 × 10^6^) cells were seeded onto 100 mm cell culture dishes and allowed to attach overnight. The next day, cells were treated with either 10 µM or 100 µM of Sirtuin inhibitors (AGK2, EX527, Sirtinol), HDAC inhibitors (SAHA, TSA) or were transiently transfected with Sirt1 and Sirt2 SiRNA KD cocktail as described before. After 48-h drug treatment or 72-h siRNA transfection, cells were collected and lysed following the protocol of Signal-Seeker Acetyl-Lysine Detection kit (Citoskeleton, Denver, CO, United States). Briefly, after the incubation period concluded, cells were washed twice with PBS and lysed in ice-cold lysis buffer, 20 mM Tris-HCl (pH 7.4), 5 mM EDTA, 150 mM NaCl, 0.5% Triton X-100, 10 mM sodium butyrate and protease/phosphatase inhibitors cocktail. Cell lysates were incubated on ice for 20 min and isolated by centrifugation at 16,000 × g for 20 min. Supernatants were recovered and protein concentration was measured as described before. 150 µg of lysate was used for the IP; 1/10 of the sample was kept as input control. Thereafter, Acetyl Lysine Affinity Beads were added to the samples and incubated at 4°C for 2 h. Beads were washed three times with the provided wash buffer and the immunoprecipitated proteins were eluted and denatured with 2 × Laemmli buffer and boiled for 5 min. Protein samples were then separated by SDS-PAGE for 1 h at 150 V. Proteins were then transferred to PVDF membranes (Bio-Rad, Hercules, CA, United States) for 2 h at 100 V and blocked for 30 min in 1×fish gelatin (Biotium Inc., Fremont, CA, United States. After blocking, membranes were incubated with rabbit monoclonal anti-NAT1 antibody (ab109114, Abcam, Cambridge, United Kingdom) and mouse monoclonal anti-β-actin, as endogenous control (A2228, Sigma-Aldrich, St. Louis, MO, United States) overnight at 4°C with gentle agitation. The next day, the membranes were washed with TBST and incubated with secondary antibodies IRDye^®^ 800 CW goat anti-rabbit and 680RD goat anti-mouse (LI-COR Biosciences, Lincoln, NE, United States) at room temperature for 1 hour. Finally, plates were washed with TBST and scanned using an Odyssey imaging system (LI-COR Biosciences, Lincoln, NE, United States). After detection of NAT1, the membranes were re-probed for acetyl-lysine-HRP detection using the antibody provided in the kit. Chemiluminescent detection was captured using a Fotodyne imaging system (Fotodyne Inc., Hartland, WI, United States). Densitometric analysis on the immunoblots was performed using ImageJ (Schneider et al., 2012) software or IMAGE STUDIO Lite software (LI-COR Biosciences, Lincoln, NE, United States), which enables quantitative analysis of blotting signals. NAT1 and acetyl-lysine levels were normalized to *β*-actin.

### Statistical Analysis

Significance levels for comparisons between groups were determined with One-way or Two-Way ANOVA, followed by Dunnett’s or Tukey’s multiple comparison tests using GraphPad Prism 9 (GraphPad Software, San Diego, CA, United States), where appropriate. For western blots and ICW, protein levels were normalized to a housekeeping protein (*β*-actin). All data were expressed as means ± standard error of the mean (SEM) of a minimum of three independent experiments. *p* values of <0.05 were considered statistically significant. The statistical parameters are specified within the figure legends.

## Results

### Deacetylase Activity Inhibition in Breast Cancer Cell Lines

**FIGURE 1:**
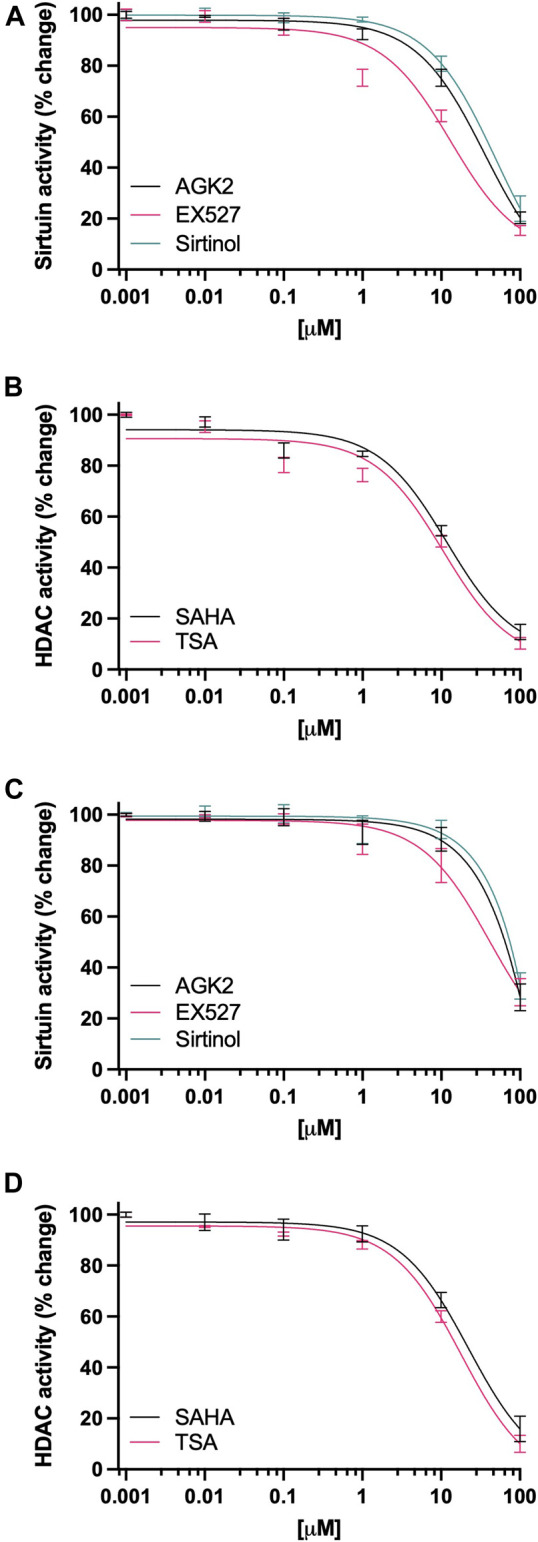
Inhibition of Sirtuin and HDAC activity in breast cancer cell lines. **(A, C)** Quantification of the catalytic activity of Sirtuin in ZR-75-1 and MDA-MB-231 cells, respectively, after 48-h treatment, cells were treated with 0–100 µM of AGK2 (black line), EX527 (pink line) or Sirtinol (green line). **(B, D)** Quantification of the catalytic activity of HDAC in ZR-75-1 and MDA-MB-231 cells, respectively, after 48-h treatment, cells were treated with 0–100 µM of SAHA (black line) or TSA (pink line). Data is presented as the mean ± SEM of four independent experiments.

MDA-MB-231 cells treated with increasing concentrations of Sirtuin inhibitors, showed a marked decrease in its deacetylase activity (Figure 1A); in the case of AGK2, 10 µM (*p* = 0.015), and 100 µM (*p* < 0.001) were significantly decreased when compared to the vehicle control (IC50 50.1 µM). For EX527, doses as low as 1 µM were significantly decreased (*p* = 0.018), 10 µM (*p* < 0.001) and 100 µM (*p* < 0.001) (IC50 40.6 µM). Sirtinol was the least potent of the group, as it significantly decreased Sirtuin activity at 100 µM (*p* < 0.001) *vs* the vehicle control (IC50 61.7 µM). On the other hand, cells treated with HDAC inhibitors (Figure 1B), SAHA significantly decreased the deacetylase activity at 10 µM (*p* < 0.001), and 100 µM (*p* < 0.001) (IC50 19.2 µM); whereas TSA inhibited deacetylase activity at 1 µM (*p* = 0.017), 10 µM (*p* < 0.001) and 100 µM (*p* < 0.001) (IC50 13.5 µM). For ZR-75-1 cells (Figure 1C), AGK2 produced inhibition at 10 µM (*p* < 0.001) and 100 µM (*p* < 0.001) (IC50 27.6 µM). EX527 inhibited deacetylase activity in doses from 1 µM (*p* < 0.001) and up (IC50 11.8 µM). Sirtinol inhibited deacetylase activity in these cells at concentrations as low as 10 µM (*p* < 0.001) (IC50 35.8 µM). The treatment with SAHA (Figure 1D), significantly inhibited the enzymatic activity in doses as low as 0.1 µM (*p* = 0.005), 1 µM (*p* = 0.001), 10 µM (*p* < 0.001) and 100 µM (*p* < 0.001) (IC50 10.9 µM). Finally, the treatment with TSA also was very effective, producing enzyme inhibition in doses as low as 0.1 µM (*p* < 0.001) and up (IC50 6.4 M).

### Effect of Deacetylase Activity Inhibition on NAT1b Message Expression

**FIGURE 2:**
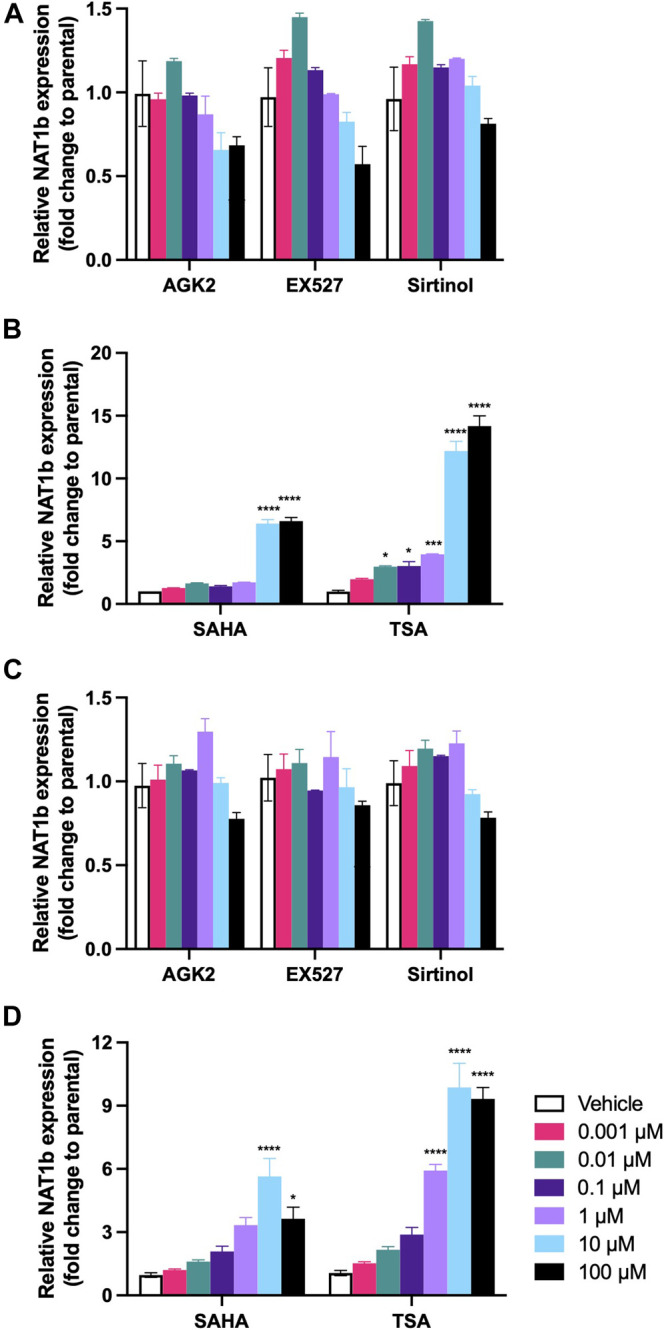
Effect of KDAC inhibition on NAT1b transcription. The relative expression of NAT1b transcript was quantified in **(A)** MDA-MB-231 cells treated with AGK2, EX527 or Sirtinol. **(B)** MDA-MB-231 cells treated with SAHA or TSA. **(C)** ZR-75-1 cells treated with AGK2, EX527 or Sirtinol. **(D)** ZR-75-1 cells treated with SAHA or TSA. Data is presented as the mean ± SEM of three independent experiments. *, *p* < 0.05; **, *p* < 0.01; ***, *p* < 0.001; ****, *p* < 0.0001 compared *vs.* vehicle control.

The effect of deacetylase activity inhibition on the levels of NAT1 mRNA expression differed following treatment with Sirtuin and HCAC inhibitors. For MDA-MB-231 cells (Figure 2A), Sirtuin inhibitors significantly decreased NAT1b transcript expression at 100 μM. AGK2 showed the greater effect (*p* < 0.001), followed by EX527 (*p* < 0.01) and Sirtinol (*p* < 0.05). On the contrary, the treatment with HDAC inhibitors (Figure 2B), resulted in more NAT1b transcript expression at 10 and 100 µM (*p* < 0.0001) for the cells treated with SAHA. The cells treated with TSA showed a dramatic increase in NAT1b transcript expression, being over 10-fold higher in cells treated with 10 µM (*p* < 0.0001) and about 13-fold higher at 100 µM (*p* < 0.0001). Similarly, in the ZR-75-1 cells (Figure 2C), the Sirtuin activity inhibition caused a significant decrease only at the highest concentration of 100 µM for EX527 (*p* < 0.001), and Sirtinol (*p* < 0.05). Finally, SAHA treatment significantly increased NAT1b transcript expression ∼5-fold at 10 µM (*p* < 0.0001) (Figure 2D), but only ∼3-fold at 100 µM (*p* < 0.05); the cells treated with TSA showed a more robust induction of NAT1b expression at 1 µM (*p* < 0.0001), 10 µM (*p* < 0.0001) and 100 µM (*p* < 0.0001).

### Inhibition of Deacetylases Modifies NAT1 Catalytic Activity

**FIGURE 3:**
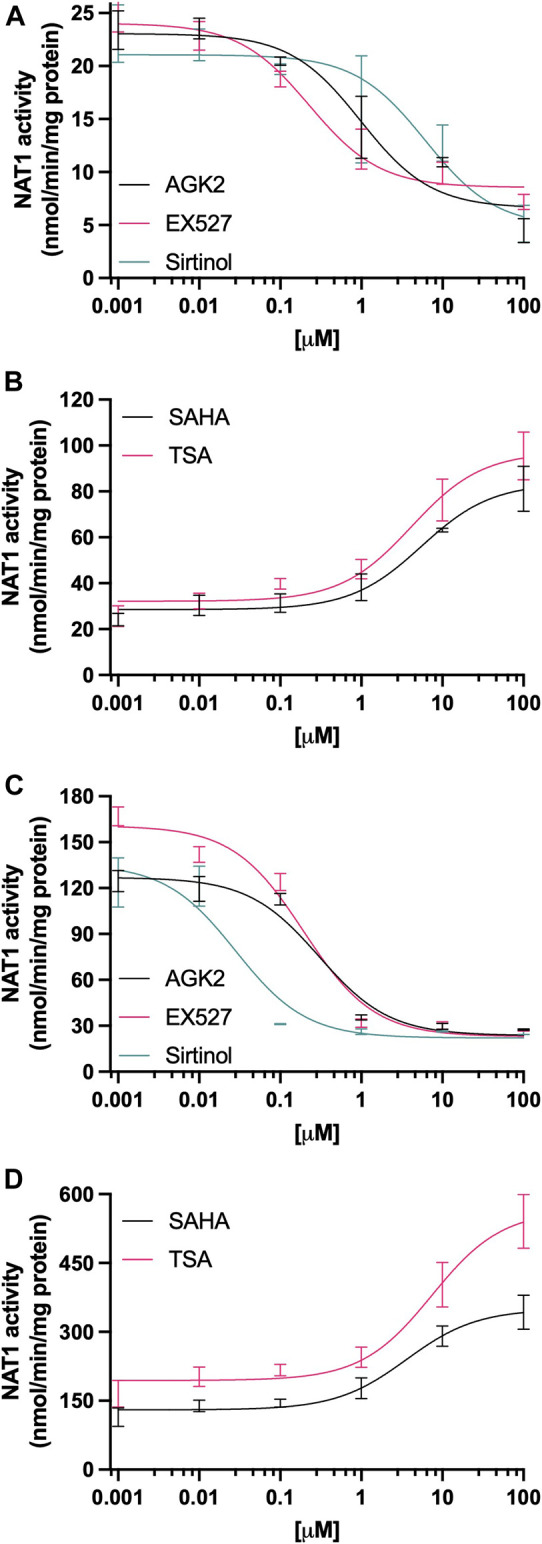
Effect of KDAC inhibition on the catalytic activity of NAT1. **(A)** MDA-MB-231 cells or **(C)** ZR-75-1 cells were treated with AGK2 (black line), EX527 (pink line) or Sirtinol (green line), then PABA *N*-acetylation activity was quantified. **(B)** MDA-MB-231 or **(D)** ZR-75-1 cells were treated with SAHA (black line) or TSA (pink line), then PABA *N-*acetylation activity was quantified. Data is presented as the mean ± SEM of four independent experiments.

The effect of deacetylase activity inhibition on the levels of NAT1 catalytic activity also differed following treatment with Sirtuin and HCAC inhibitors in MDA-MB-231 cells, the SIRT2 inhibitor AGK2 (Figure 3A), significantly decreased NAT1 catalytic activity in concentrations as low as 1 µM (*p* < 0.0001, *K*_*i*_ (10.85 µM)). SIRT1 inhibitor EX527 showed the same effect in concentrations as low as 0.1 µM (*p* < 0.01, *K*_*i*_ (7.03 µM)); whereas SIRT1 and two inhibitor Sirtinol reduced NAT1 activity at 1 µM (*p* < 0.001, *K*_*i*_ (21.85 µM)). In contrast, treatment with HDAC activity inhibitor SAHA (Figure 3B), induced an upregulation in NAT1 *N*-acetylation of PABA when treated with 10 and 100 µM (*p* < 0.0001). TSA also induced an increase in *N*-acetylation of PABA in these cells when treated with 1 µM (*p* < 0.01), 10 µM (*p* < 0.0001) and up. We observed a similar response in ZR-75-1 cells (Figure 3C), where AGK2 produced a marked decrease in NAT1 when treated with at least 1 µM [*p* < 0.0001, *K*_*i*_ (0.55 µM)], EX527 showed a very potent effect in these cells in doses as low as 0.01 µM (*p* < 0.001, *K*_*i*_ (0.39 µM)), and Sirtinol downregulated NAT1 activity at 0.1 µM (*p* < 0.0001, *K*_*i*_ (0.02 µM)) and up. In contrast, after treatment with HDAC inhibitors (Figure 3D), NAT1 catalytic activity was upregulated by SAHA in concentrations from 10 µM (*p* < 0.0001), and TSA from 1 µM (*p* < 0.05).

### Sirtuin Knockdown Efficiency

**FIGURE 4:**
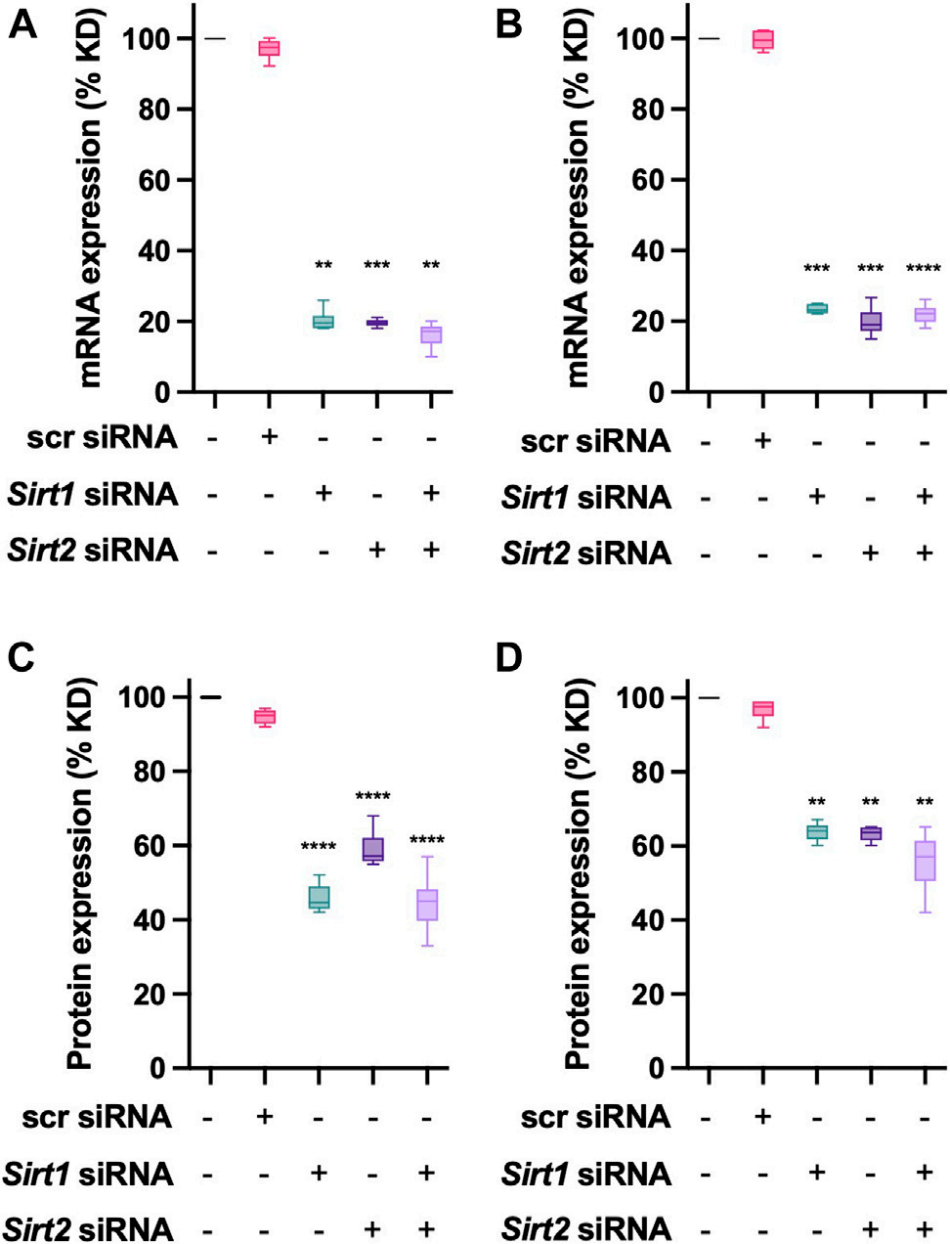
*Sirt1* and *Sirt1* knockdown in breast cancer cells. **(A)** MDA-MB-231 cells or **(B)** ZR-75-1 cells were transfected with control (*scrambled*), *Sirt1* or *Sirt2* siRNAs. Then the relative expression of the target genes was evaluated after a 48-h transfection. **(C)** MDA-MB-231 or **(D)** ZR-75-1 cells, were transfected for 72-h, at that time the protein expression of the target protein was evaluated. Data is presented as the mean ± SEM of three independent experiments. **, *p* < 0.01; ***, *p* < 0.001; ****, *p* < 0.0001 compared *vs. scr* control.

To further evaluate the effect of Sirtuin deacetylases on NAT1, transient transfections using siRNA were performed. To avoid off-target effects from the siRNA, a mix of three different sequences for *Sirt1* and *Sirt2* (Table 2) were used. Individual transfections were done to obtain the optimal concentrations (data not shown), and the final experimental conditions were performed as described in the methods section. Target mRNA expression was quantified 48-h post-transfection. We observed that for MDA-MB-231 cells (Figure 4A), we obtained an 80% KD efficiency (*p* < 0.01) for *Sirt1* and *Sirt2* (*p* < 0.001), double KD cells showed an 85% KD efficiency (*p* < 0.01), when compared to the scrambled siRNA. In the case of ZR-75-1 cells (Figure 4B), a very robust KD efficiency was achieved under the conditions tested; *Sirt1* (*p* < 0.001), *Sirt2* (*p* < 0.001) and *Sirt1*+*Sirt2* (*p* < 0.0001), with at least a 77% KD for these targets. After 72 h post-transfection, the protein expression of the target gene was quantified as previously described. For MDA-MB-231 (Figure 4C), siRNA transfection, produced a robust downregulation (*p* < 0.0001) of the protein level for *Sirt1*(55% KD), *Sirt2* (40% KD) and *Sirt1*+*Sirt2* (60% KD). ZR-75-1 cells (Figure 4D) exhibited downregulation of 35% for *Sirt1* (*p* < 0.01), *Sirt2* 35% KD (*p* < 0.01) and 42% KD for the double KD (*p* < 0.01). Further experiments evaluating the effect of transient transfection on NAT1 were carried out with the same transfection conditions.

### Effect *Sirt1* and/or *Sirt2* KD on NAT1 Expression and *N*-Acetylation Activity

**FIGURE 5:**
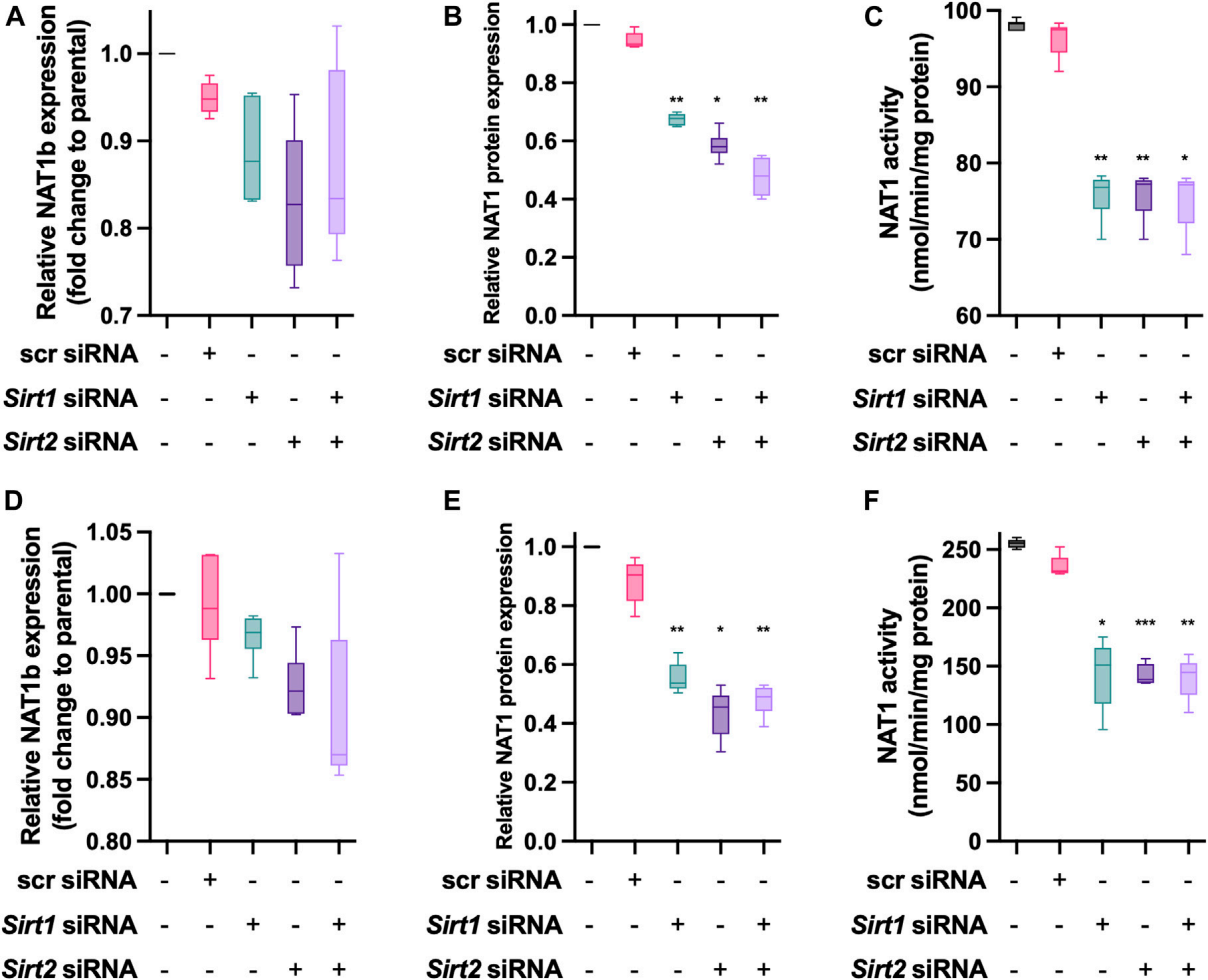
Effect of *Sirt1* and *Sirt2* KD on NAT1 expression and activity in MDA-MB-231 and ZR-75**-**1 breast cancer cells. MDA-MB-231 cells were transfected with *Sirt1* and *Sirt2* siRNAs for 72-h, then **(A)** NAT1b transcript. **(B)** NAT1 protein expression and **(C)** NAT1catalytic activity was quantified. ZR-75-1 cells were transfected with *Sirt1* and *Sirt2* siRNAs for 72-h, then **(D)** NAT1b transcript **(E)** NAT1 protein expression and **(F)** NAT1 catalytic activity was quantified. Data is presented as the mean ± SEM of four independent experiments. *, *p* < 0.05; **, *p* < 0.01 compared *vs. scramble* control.

MDA-MD-231 cells transiently transfected with siRNA targeting *Sirt1*, *Sirt2* or both (Figure 5A) did not result in significant changes in the expression of NAT1b transcript. Then, we quantified NAT1 protein expression in the transfected cells (Figure 5B). *Sirt1* KD produced a 30% decrease (*p* < 0.01) and *Sirt2* KD produced a 40% decrease of NAT1 protein (*p* < 0.05). The double *Sirt1*/*Sirt2* KD resulted in a 50% loss of NAT1 protein present in the cells (*p* < 0.01). NAT1 *N*-acetylation activity also was significantly decreased in the transfected cells (Figure 5C), individual *Sirt1* and *Sirt2* KD produced ∼20% loss of NAT1 catalytic activity (*p* < 0.01) and Sirt1/Sirt2 KD cells showed a similar loss in NAT1 function (*p* < 0.05). For the ZR-75-1 cells, no changes were observed in NAT1b transcript expression in any of the experimental conditions (Figure 5D). KD of *Sirt1* caused a 45% decrease in NAT1 protein amount (Figure 5E (*p* < 0.01). Sirt2 KD caused the loss of 58% NAT1 protein (*p* < 0.05); whereas the Sirt1/Sirt2 KD cells caused 55% loss (*p* < 0.01). Finally, NAT1 catalytic activity was also decreased by the KD in this cell line (Figure 5F). *Sirt1* KD caused a 44% loss of function of NAT1 (*p* < 0.05). *Sirt2* KD produced a very similar result (*p* < 0.001). *Sirt1*/*Sirt2* KD cells caused a 48% loss of NAT1 function on these cells (*p* < 0.01).

### Acetylation Status of NAT1

**FIGURE 6:**
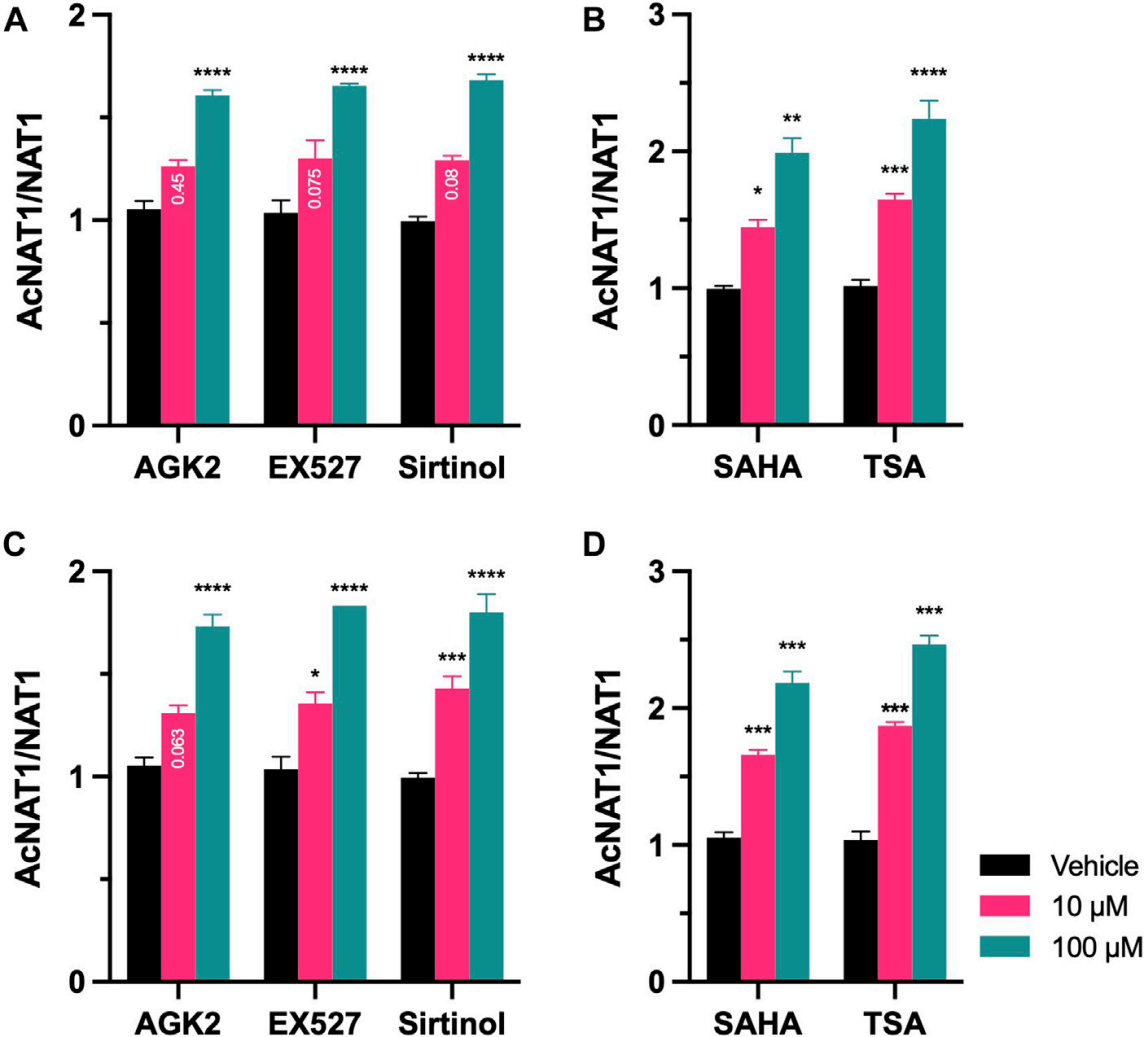
Acetylation status of NAT1 after treatment with KDAC inhibitors. **(A)** MDA-MB-231 or **(C)** ZR-75-1 cells were treated with AGK2, EX527 or Sirtinol for 48-h, then ac-Lysine was immunoprecipitated and probed for NAT1 protein expression. **(B)** MDA-MB-231 or **(D)** ZR-75-1 cells were treated with SAHA or TSA for 48-h, then ac-Lysine was immunoprecipitated and probed for NAT1 protein expression. Data represents the ratio of AcNAT1/NAT1 abundance. Data is presented as the mean ± SEM of three independent experiments. *, *p* < 0.05; **, *p* < 0.01; ***, *p* < 0.001; ****, *p* < 0.0001 compared *vs.* vehicle control.

NAT1 acetylation status was evaluated following inhibition of Sirtuin and HDAC proteins. After 48-h treatment, Ac-lysine in NAT1 was quantified and normalized to total NAT1 present in the cells. For MDA-MB-231 cells, treatment with Sirtuin inhibitors (Figure 6A), increased the amount of acetylated Lysine residues of NAT1 in the presence of 100 µM of AGK2 (*p* < 0.0001), 100 µM of EX527 (*p* < 0.0001), and 100 µM of Sirtinol (*p* < 0.0001). The same cell line, treated with HDAC inhibitors (Figure 6B), showed that 10 µM of SAHA increased NAT1 acetylation by 1.45-fold (*p* < 0.05), whereas 100 µM of the drug resulted in a 2-fold increase of the Lysine residues of the protein being acetylated (*p* < 0.01). TSA increased the acetylation of Lysine residues by 1.6-fold (*p* < 0.001) at 10 μM, and 2.1-fold at 100 µM (*p* < 0.0001). The same response pattern was observed on ZR-75-1 cells (Figure 6C), the presence of AGK2, increased the acetylation of NAT1 lysine residues at 100 µM (*p* < 0.0001), Sirtuin two inhibition by EX527, had a similar effect on NAT1 acetylation status at 10 µM (*p* < 0.05), and 100 µM (*p* < 0.0001). Sirtuin one and two inhibitions by Sirtinol increased the acetylation status at 10 µM (*p* < 0.001) and 100 µM (*p* < 0.0001). Finally, the inhibition HDAC proteins (Figure 6D), by SAHA induced a 1.6-fold increase in NAT1 acetylation status compared to the vehicle control at 10 µM (*p* < 0.001), and 2.1-fold at 100 µM (*p* < 0.0001). Treatment with TSA produced a 1.8-fold increase in the acetylation status of NAT1 (*p* < 0.001), and a 2.4-fold increase in NAT1 acetylation at the highest dose of 100 µM (*p* < 0.001).

## Discussion

We investigated the effect that Lysine-acetylation, as a post-translational modification (PTM), has on the catalytic activity of NAT1 in two different breast cancer cell lines, MDA-MB-231 and ZR-75-1. Several different studies have demonstrated that NAT1 is overexpressed in breast cancer, suggesting that it plays an important role in breast cancer development and growth (Tiang et al., 2010; Tiang et al., 2011; Carlisle and Hein, 2018; Zhang et al., 2018). Recent reports have established that *N*-acetyltransferase enzymes are a target of regulation by acetylation (Salazar-Gonzalez et al., 2018a; Turijan-Espinoza et al., 2018; Butcher et al., 2020). Furthermore, K^100^ and K^188^ have been identified as important sites for this regulatory process (Minchin and Butcher, 2015; Butcher et al., 2020). Based on the proximity of these Lysine residues with the catalytic pocket of NAT1, the authors proposed these sites as the main target for Lysine acetylation. Acetylation is generated by KAT-catalyzed transfer of an acetyl group from acetyl-CoA to the ɛ-amino side chain of Lysine and is reversed by KDACs. Recent studies show that acetylation also occurs through non-enzymatic mechanisms and is affected by the availability of acetyl-CoA. In our experiments we tested the effect inhibition of KDAC proteins like Sirtuin (AGK2, EX527, Sirtinol) or HDAC proteins (SAHA, TSA).

Our results show that the activity of these proteins can be abolished almost completely in MDA-MB-231 and ZR-75-1 breast cancer cell lines (see Figure 1). In our experiments, we were able to produce significant decrease of KDAC proteins; the most effective of the inhibitors we used was EX527, a highly selective and potent SIRT1 inhibitor, produced a very robust inhibition at 1 µM. Followed by the selective SIRT2 inhibitor AGK2, and Sirtinol (SIRT1 and SIRT2 inhibitor), which were effective at doses of 10 µM. In the case of HDAC inhibitors, TSA produced a very robust effect in these cell lines in concentrations of 0.1 µM. On the other hand, SAHA also induced an inhibition of enzymatic activity in doses as low as 1 µM. No significant changes in cell viability were observed in these experiments. After we observed this inhibition, we proceeded with the study of NAT1b transcript expression in these cell lines after the inhibition of Sirtuin or HDAC activity. For both cell lines, the inhibition of Sirtuin activity failed to produce a significant change in the transcription of NAT1b (see Figure 2), however, the inhibition of HDAC activity, produced a striking upregulation of NAT1b transcript expression; for cells treated with SAHA, a 4-6-fold upregulation of NAT1b transcription in doses of 10 and 100 µM was observed. When we repeated the experiment, using the HDAC inhibitor TSA, the upregulation of NAT1b transcription was even more robust, producing a significant increase in doses as low as 0.01 µM and 3-fold increase in transcription, all the way to a 12-fold increase in the transcription of NAT1b at 100 µM in MDA-MB-231 cells. In the case of ZR-75-1 cell line, the upregulation of NAT1b message expression was significant in doses as low as 1 µM (6-fold increase), with greatest increase at 100 µM resulting in a 9-fold upregulation in NAT1b transcription.

One of the endpoints of our study was the evaluation of KDAC inhibition on NAT1 catalytic activity. We observed that the inhibition of Sirtuin activity, had a significant impact on the *N*-acetylation of PABA (see Figure 3), SIRT1 and SIRT2 inhibition produced a decrease in *N*-acetylation, whereas the treatment with HDAC inhibitors showed the opposite effect, showing a dose-dependent increase in the *N*-acetylation of PABA, reaching up to a 3-fold increase at the highest doses. We aimed to confirm the findings about Sirtuin downregulation of NAT1 by transfecting the cells with specific siRNAs targeting *Sirt1* or *Sirt2* (see Figure 5), we observed the same effect, KD of *Sirt1/2* genes produced a significant decrease in the *N*-acetylation capacity of NAT1. Furthermore, when we evaluated NAT1 protein expression, we also observed a decrease in NAT1 protein, with no significant changes in NAT1b transcript expression. These findings are consistent with previous studies (Butcher et al., 2020), where the authors describe the role of SIRT1 and SIRT2 in the acetylation state of NAT1. In this study, the authors describe how the deacetylated form of NAT1 has a higher *V*_max_ towards PABA, compared to the acetylated form of the protein; no effect was observed in the substrate affinity (*K*_M_) between the acetylated/deacetylated form of the protein. Since the K^100^ and K^188^ are conserved between NAT1 and NAT2, it might be possible that, the acetylation state of NAT2 would be regulated by SIRT1 and SIRT2 proteins, as described in (Salazar-Gonzalez et al., 2018a; Turijan-Espinoza et al., 2018), where *N-*acetylation capacity of these proteins in blood mononuclear cells was regulated by Sirtuins.

We also evaluated the role of HDAC proteins in the acetylation status of PABA. Our data shows how the inhibition of these proteins upregulates the transcription of NAT1b (see Figure 2). Acetylation of Lysine residues on histone tails generates positive charges, which will neutralize the negatively charged DNA, thus unwinding the heterochromatin. This process can increase the pore space of chromatin, ensuring sufficient space for the transcription machinery to initiate and elongate products (Gorisch et al., 2005; Shogren-Knaak et al., 2006; Wang et al., 2009). Although HDACs generally function as gene silencers, they can activate transcription (Kurdistani et al., 2002; Wang et al., 2002). Histone deacetylation and DNA methylation at CpG islands constitute another layer of epigenetic regulation of gene expression; methylation of the 5′-untranslated region of NAT1 has been linked associated to tamoxifen-resistant breast cancer tumors (Kim et al., 2010). Different studies demonstrated that the 3′-untranslated region of NAT1 is a direct target for miR-1290 (Endo et al., 2014), and miR-6744-5p (Malagobadan et al., 2020), where its expression is downregulated, promoting distant metastasis in triple-negative breast cancers (Butcher and Minchin, 2012). In our study, we observed a marked increase in the *N*-acetylation activity of NAT1 after the treatment with HDAC inhibitors, consistent with the increase in NAT1b transcription. The same phenomenon was observed by Paterson and colleagues (Paterson et al., 2011); the authors describe an upregulation of NAT1b transcription, as well as an increase in NAT1 catalytic activity, after treatment with TSA. This is explained by the recruitment of Sp1 by TSA, probably because of the relaxed chromatin surrounding the NAT1b promoter region.

The disregulation of epigenetic control in cells has been noted as a common charactetistic of cancerous tumor cells. Therefore, the role of epigenetic drugs has become increasingly important in reverting the malignant phenotype. HDACs and their inhibitors have become more influential in epigenetics as they provide specific treatments to target specific forms of cancer (Lin et al., 2006; Barneda-Zahonero and Parra, 2012; Bai et al., 2019). Acetylation neutralizes the positively charged histone Lysine residues, causing a relaxed chromatin conformation that increases the access of transcriptional modifiers to the gene. In the removal of the acetyl group from a histone, chromatin condensation is induced, leading to transcription repression (Hassell, 2019; Li et al., 2020). These inhibitors play a major role in several biological processes, like cell cycle progression, proliferation and differentiation (Garcia-Martinez et al., 2021). Further studies might be of interest to understand interplay between the mechanism of the NAT1 transcription regulation after the inhibition of HDAC and its apparent synergistic effect with the epigenetic changes caused by these inhibitors, potentially leading to new therapeutic targets and clinical applications.

Finally, the acetylation status of NAT1 was evaluated (see Figure 6). We found that in the presence of Sirtuin inhibitors and HDAC inhibitors, the acetylated form of the protein is more prevalent, compared to the untreated cells. In the case of SIRT1 and SIRT2, these results are consistent with previous findings (Butcher et al., 2020), where the authors demonstrated the influence of the acetylation of K^100^ and K^188^ in the catalytic activity of NAT1. In the afore mentioned study (Butcher et al., 2020), an equilibrium between the acetylated/deacetylated forms of NAT1 is present in the cell, however this equilibrium might be affected by the variations in the proteins responsible of such balance, p300/CBP and SIRT1/SIRT2. This is consistent with our *N*-acetylation activity data; in cells where the Sirtuin activity was inhibited, the acetylated form of the protein should be more prevalent, thus leading to a decrease in *N*-acetylation activity of NAT1. This was confirmed in our experiments by measuring the ratio between the acetylated and non-acetylated form of the protein. On the other hand, we observed that inhibition of HDAC activity resulted in the acetylated form of the protein to be more prevalent. Thus, the increase in NAT1 catalytic activity observed following treatment with HDAC inhibitors suggests that the increase in NAT1b transcript is more important than the prevalence of the acetylated form of NAT1.

In several preclinical studies and clinical trials, the use of HDAC and Sirtuin inhibitors has been demonstrated as a promising powerful therapeutic alternative in various cancers (Guerriero et al., 2017). These inhibitors can significantly attenuate tumor burden by limiting tumor growth and metastasis. These compounds can also induce DNA damage, cell cycle arrest, apoptosis, and autophagy to promote cancer cell death (Carafa et al., 2018; De et al., 2018; Carafa et al., 2019; Tae et al., 2020). Several studies have shown that NAT1 is upregulated in cancer cells (Carlisle and Hein, 2018; Zhang et al., 2018; Stepp et al., 2019), the results of the present study show that the acetylation status of NAT1 is an important factor that might have a relevant role in the progression of cancer. Furthermore, understanding of NAT1 regulation in breast cancer can be potentially applied to development of new therapies using small molecules like the ones presented here.

In conclusion, the present study shows that NAT1 catalytic activity is influenced by the acetylation status of the protein. The inhibition of KDAC activity resulted in modifications in the catalytic response of NAT1. Our data shows that SIRT1 and SIRT2 are responsible for this regulatory effect, as well as histone deacetylases (HDAC). Additional studies are needed to understand this regulation mechanism and develop an application that can help therapies for cancer patients.

## Data Availability

The raw data supporting the conclusion of this article will be made available by the authors, without undue reservation.
